# Behaviours Expressed by Rodeo Calves during Different Phases of Roping

**DOI:** 10.3390/ani13030343

**Published:** 2023-01-19

**Authors:** Sylvia Dixon, Di Evans, Thinza Vindevoghel, Michael P. Ward, Anne Quain

**Affiliations:** 1 School of Veterinary Science, University of Sydney, Camperdown, NSW 2006, Australia; 2 RSPCA Australia, Deakin, Canberra, ACT 2600, Australia; 3 Independent Researcher, Perth, WA 6164, Australia

**Keywords:** calf roping, rope-and-tie, rodeo, ethogram, animal welfare, animal behaviour

## Abstract

**Simple Summary:**

Calf roping is a rodeo event that involves the release, pursuit, lassoing and leg-tying of a calf by a rider on horseback. Welfare concerns for calves used in these events include distress and potential physical injuries. This study evaluated video footage of calf roping (also known as rope-and-tie) from two rodeos and assessed calves’ behaviour to identify any signs of distress during five different phases of roping. Calves in the study showed signs of distress and fear across all stages of roping. These findings suggest that calves experience fear and distress in calf roping events, which can inform discussions of the welfare of calves used in rodeos.

**Abstract:**

There are significant welfare concerns with the use of calves in calf roping (also known as rope-and-tie) events in rodeos. However, little work has been carried out to scientifically assess calves’ behavioural responses to the stressors of these events. This study evaluated video footage of calves (*n* = 15) during roping events from two rodeos. An ethogram was created to assess behavioural parameters during five phases of a calf roping event: Chase, Lasso, Catch and Restraint, Leg Tie and Release. Six behavioural parameters were observed during each of the five phases: Ear position (Axial or Back Up/Down), Eye White, Leg Movement (Slow/Fast), Tail Position (Rigid/Swish/Relaxed), Vocalisation and Evasive Behaviour. The presence or absence of each behaviour during each phase of roping was documented. Chi-squared analysis found significant (*p* < 0.001) differences in the proportions of behaviours observed in all five phases of the event. Binary logistic regression was performed, and six behaviours were observed to be significant across all phases: Evasive Behaviour (*p* < 0.001), Vocalisation (*p* = 0.002) and Legs Fast (*p* = 0.016) were more likely to be observed in comparison to Ears Axial, which was used as a reference category. Eye White (*p* < 0.001), Legs Slow (*p* < 0.001) and Tail Relaxed (*p* < 0.001) were less likely to be observed than Ears Axial. This study found that calves exhibit signs of distress in all of the phases of calf roping, including the Release phase.

## 1. Introduction

Rodeo, a Spanish word meaning “round up”, is a competitive sport thought to have originated from early Spanish settlers in the USA [1]. Subsequently, competitions arose to showcase the skills of stockpersons [2,3]. The first organised rodeo event, the Prescott Frontier Days Rodeo, was held on 4 July 1888 in Prescott, Arizona [1]. With the gradual replacement of horses with motor vehicles for ranching, rodeos evolved into a sporting competition and a form of entertainment. One of the largest rodeos is the Calgary Stampede in Canada, which attracts over one million people each year [4]. However, there is an increasing public sensitivity to the use of animals in rodeos. Ethicist Professor Bernard Rollin identified rodeos—particularly calf roping—as a key example of “problematic animal use” which degrades the moral status of animals [5,6].

In Australia, Canada and the United States of America, the most common events at rodeos are saddle bronc riding, bareback bronc riding, bull riding, steer wrestling, team roping and calf roping (or rope-and-tie) [7,8]. The first calf roping event was held at a rodeo in Prescott in 1917 [1,2]. Calf roping is particularly contentious due to concerns for the welfare of calves, who are handled during the competition in ways that cause distress, fear, and the risk of injuries or even death throughout the various stages of the event [9,10].

Calf roping originated from the need to capture calves for procedures, such as castration or dehorning, but has become an event for public entertainment and sport [2]. It involves a competitor on horseback chasing a calf (approximately 100–125 kg in body weight and 3–4 months of age) who is released from a holding chute. The competitor aims to lasso the calf around the neck with a rope while on horseback, dismount, run to the end of the rope, throw the calf onto their side and tie three of their legs together [2,11]. The rider’s time is noted once completion of the tie is achieved, and it has been demonstrated that the calf is securely restrained. Competitors are rewarded for speed, with horses starting from a gallop as the rider chases, ropes and ties down the calf in the shortest possible time. Under the rules, the calf must be stopped by the rope, but should not fall [11]. Ideally, rodeo horses are trained to back up, if necessary, to keep tension on the rope attached to the calf while the rider dismounts and ties the calf. The rider then remounts the horse and slackens the rope, after which the tie on the calf must hold for at least six seconds. If the calf falls during roping, the competitor must stand the animal up before throwing them to the ground by hand. Competitors maximise their speed by having assistants push calves out of the holding chute. The maximum time limit for each competitor is 30 seconds. If the judges rule that a calf was pulled backwards off their feet, abruptly thrown sideways or stopped abruptly, the competitor may be disqualified [11,12]. Individual calves can be used for roping up to three times in one day at rodeo competitions and they can be used at multiple events over a season. In addition, calves are used at rodeo schools which, in most jurisdictions, have no standards to restrict the number of times they are used in a defined period of time. Thus, these young calves can be subjected to this treatment repeatedly.

Supporters of rodeos argue that calf roping is an art, and an exhibition of horse-riding skills that has significant historical and cultural value [12,13]. Rodeo has been portrayed in popular cinema. For example, the revered and skilled rodeo competitor is portrayed in movie productions, such as *Walk. Ride. Rodeo.* (2019) and *Cowboy Up,* (2001) [14].

Critics of rodeos, including animal advocacy organisations, such as RSPCA Australia and Animal Liberation, have raised concerns over what they describe as unnecessary suffering due to fear and distress experienced by calves used in rope-and-tie events, as well as potential physical injuries, particularly when being roped and thrown to the ground [15,16]. Injuries may include damage to the trachea and soft tissues of the neck due to jerking of the rope, choking from being dragged whilst roped, and rib fractures and bruising when calves are thrown to the ground [7,9,15]. These injuries may be severe enough to necessitate euthanasia of affected calves [17,18]. As calves are prey species, they may not display obvious signs of pain when injured in an attempt to avoid predation [19,20,21]. Masking of pain when in the presence of a predator makes ruminants’ pain thresholds appear higher, therefore these findings have significant implications when assessing prey pain perception in the presence of a predator [22].

Given the nature of calf roping—involving lassoing causing the calf to be halted abruptly due to significant tension on the neck—deceleration injuries, as well as injuries specifically associated with strangulation, are considered to be significant risks [23]. Cervical radiographs of rodeo calves have not shown evidence of injury or abnormalities [24]. However, radiographic findings of whiplash injuries, which are an important cause of chronic disability in humans, were also unremarkable as the injuries occur to soft tissue structures [25].

Deceleration injuries are a type of motion injury where the body forcibly stops but the contents of body cavities remain in motion due to inertia. The brain is particularly vulnerable and prone to injury with this type of trauma, with shoulders and the neck also being affected, especially where an abrupt halt at high speed is experienced (e.g., vehicle accidents) [26]. Deceleration injuries are also reported in human sports where athletes running at high speed stop or change direction suddenly [26]. 

Strangulation injuries occur due to a mechanical force being applied to the neck and surrounding structures. In humans, external signs of injury may be minimal or not apparent and may not reflect the extent of damage to underlying tissues [27]. In animals, even external injuries are more difficult to determine due to the presence of a hair coat and in many cases a dark coat colour. Calf roping has been described by Dr Hugh Wirth, veterinarian and former president of RSPCA Victoria, as a form of “horizontal hanging” [28].

Despite conflicting views about calf roping events, there is a growing body of literature demonstrating the negative impacts of rodeo events on the behaviours and emotional states of animals including calves [7,29,30]. Sinclair and colleagues found that calves used in roping events experience physiological stress [29]. Blood samples from experienced calves (calves who have been used in rodeos) before and after a roping event showed higher levels of serum cortisol, epinephrine and norepinephrine post-roping compared to pre-roping. Increased levels of stress hormones were also evident in naïve calves (calves who have not been used in rodeo) after marshalling across the arena by a rider on horseback. These results showed that rodeo events are stressful for both experienced and naïve calves [29].

A review by Temple Grandin examined the behavioural responses to handling, genetics, adaptation to handling, novelty and aversion tests on different species including cattle. Animals who have been habituated to a procedure in a manner that is low-stress may have low baseline cortisol levels. However, calves who were restrained had elevated cortisol levels (24 ng/mL as opposed to the baseline of 6 ng/mL) and displayed stress-related behaviours including vocalisation and escape attempts [31].

Qualitative behaviour assessments (QBA) examining still images suggest that calves in roping events exhibit different emotions and behaviours depending on whether they are being chased by the rider on horseback compared to when they have been released after the ropes are removed [7]. When calves were pursued on horseback, their behaviour was consistent with distress and fear, whereas calves who had been released after roping to exit the arena showed signs of being calm and relieved. These findings suggest that rodeo events are potentially distressing to calves used in these events. 

The aim of this project was to determine whether calves in rodeos exhibit signs of distress. We hypothesized that video footage could be used to identify the behaviours expressed by calves in calf roping events. Additionally, we hypothesised that calves would display emotions associated with negative affective states during the first four phases of the event while calves would display fewer behavioural signs of distress in the Release phase.

## 2. Materials and Methods

### 2.1. Data Collection

This retrospective study utilized footage provided by RSPCA Australia of two calf-roping events that took place in Queensland, Australia, in 2021. Video footage of 15 calves used in rope-and-tie events were analysed.

To facilitate analysis, the calf roping event was divided into five phases: Chase, Lasso, Catch and Restraint, Leg Tie, and Release (see Table 1). Mis-roping was characterised as the rider lassoing the calf around the legs or part of the body other than the neck. Behaviours were analysed and compared for all five phases.

Viewing video footage of the 15 calves also allowed for observations of the physical impacts on the calves for these phases (Table 2). These include chasing, sudden deceleration, choking, restraint, throwing/dropping to the ground and tying legs. Analysing physical and behavioural impacts may reveal correlations between different physical impacts and the behavioural responses displayed by calves.

### 2.2. Ethogram and Scoring Sheet

An ethogram developed for this project, which identified the behaviours expressed by calves in a calf roping event, formed the basis for the scoring sheet used to record the absence or presence of these behaviours (Table 3). The behaviours were: Ear position (Axial or Back), Leg Movement (Fast or Slow), Eye White, Tail Position (Rigid, Swish, Relaxed), Vocalisation and Evasive Behaviour. These behaviours have previously been associated with positive and negative affective states in calves [7,29]. Ears Back, Legs Fast, Tail Rigid, Eye White, Vocalisation and Evasive Behaviour indicate negative affect states in calves, whereas Ears Axial and Legs Slow are associated with positive affect states.

The videos were viewed and scored independently by two authors (SD and TV). For each phase of the event, the presence of a behaviour was allocated the score of 1, while the absence of a behaviour was scored 0. If a behaviour could not be assessed, for example leg movement could not be analysed during leg tying, the code N/A (not applicable) was recorded. Some phases could not be scored due to obstructed views, so these were allocated the score −99 to denote missing data. The scores were also compared to identify any variations in the dataset prior to analysis. 

The scores were compared to identify any points of disagreement between the authors scoring the videos. Where these scores differed, the footage was reviewed by the two authors and discussed until an agreement was reached for all the data points. 

The two authors also recorded notes of any observations for each of the calves that included any relevant behaviours displayed by the calves or the competitors in the event. 

### 2.3. Data Analysis

Data were uploaded into SPSS (IBM SPSS Statistics v28, IBM Corporation) to facilitate statistical analysis.

Contingency tables and chi-squared tests were performed to analyse behaviours of calves in which each phase was analysed separately.

Binary logistic regression was used to build a model to investigate whether there was a significant association between behaviour and status across all phases of the event. The binary outcome was status, denoted as present (“1”) versus absent (“0”). Each calf was fitted as a fixed effect. In addition, location was evaluated as a confounder of the associations.

## 3. Results

Video footage of 15 calves from both rodeos was analysed. A total of 750 observations were made by each observer based on these 15 calves, 5 phases and 10 behaviours. The total number of possible behaviours expressed varied depending on the phase; due to the nature of the phases, some behaviours could not be assessed, i.e., leg movement during the Leg Tie phase.

### 3.1. Statistical Analysis 

Cross tabulation analysis of the data showed Evasive Behaviour to be the most commonly observed behaviour across the whole event; it was displayed by the calves a total of 59 times. Legs Slow was the least-observed behaviour, displayed a total of 10 times. Eye White was observed predominately in Catch and Restraint and Leg Tie phases, and Vocalisation was observed the most in the Catch and Restraint phase. Ears Axial and Ears Back showed an inverse relationship: Ears Axial was observed most commonly in the later phases of the event, whereas Ears Back was observed early in the event during Chase and Lasso. Tail Rigid was observed the most in the first four phases and was displayed the least once the calf was untied and released. Conversely, Tail Relaxed was observed most in the Release phase.

Chi-squared tests were performed on each of the phases of the event. Significant (*p* < 0.001) differences in the proportions of behaviours were observed in all five phases of the event (Table 4).

The associations between behaviour and phase were investigated to determine which behaviours were most likely to be observed across the five phases of the rope-and-tie event. Binary logistic regression was used to create a model in which status “1” was used as the outcome variable for each calf.

Behaviour and phase were studied whilst controlling for calf as a fixed variable. There was no significant association between the behaviours observed and event phase; however, across all phases, some behaviours were identified which were more or less likely to be observed (*p* < 0.001, Table 5). In this analysis, Ears Axial was used as the reference category and six significant behaviours were identified: there were increased odds of Evasive Behaviour (3.74 times) and Legs Fast (2.583 times) being observed, and reduced odds of Eye White (0.168 times), Vocalisation (0.342 times), Legs Slow (0.157 times) and Tail Relaxed (0.202 times) being observed.

### 3.2. Observations

**Figure 1:**
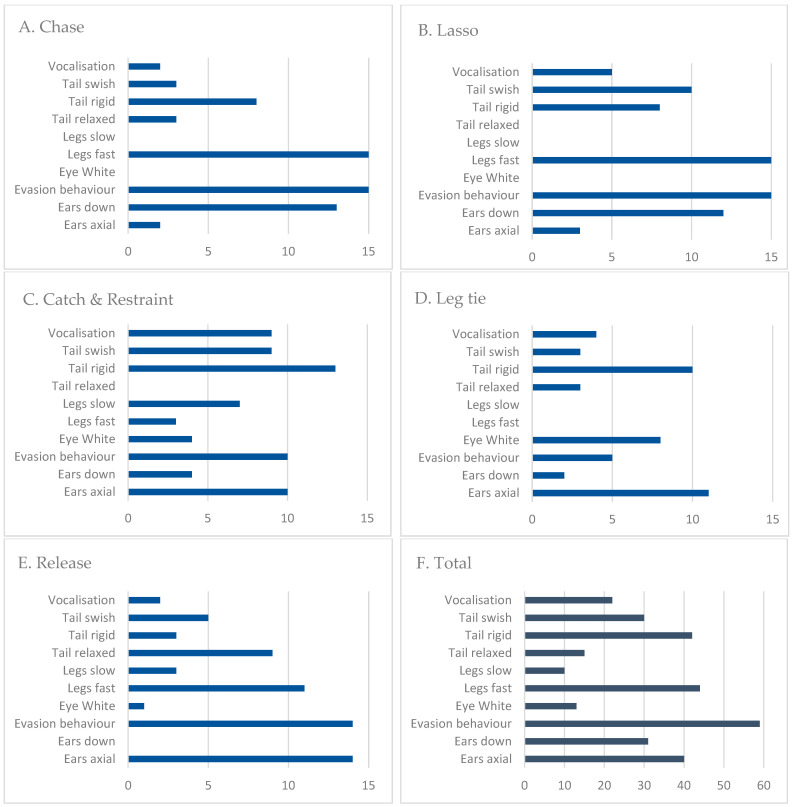
Column graphs (**A**–**F**) display the cross-tabulation results of the behaviours expressed by calves (*n* = 15) in calf roping events. Each graph represents one phase of the event (**A**–**E**), and graph (**F**) displays the total number of behaviours expressed by calves across the phases of events.

Despite Evasive Behaviour being the most prominent behaviour found in the study (Figure 1F), six calves did not resist being held down; instead, they lay still or lay their heads on the ground during the Leg Tie phase.

Legs Fast was frequently observed in four out of the five phases of the event. Generally, the calves did not walk or trot to the exit slowly, but rather ran away from the competitor towards the exit upon their release.

Eye White and Vocalisation were less frequently observed across all phases of the event compared to other behaviours, such as Evasive Behaviour or Legs Fast (Figure 1F). However, these behaviours were observed predominantly in the Catch and Restraint and Leg Tie phases when the calves were being handled by the competitor. Several calves displayed both prominent Eye White and Vocalisation during these phases, including one calf who showed mouth gaping and tongue protrusion during Lassoing and Restraint. 

In some footage, a stock assistant could be seen standing next to the chute pushing and shoving calves against the chute prior to the commencement of the event. The aim of pushing and shoving calves appeared to be to ensure that they ran out the chute as quickly as possible when released. This is permitted under the rodeo event rules [11].

## 4. Discussion

Behaviours associated with negative affective states were found in all phases of calf roping, including the Release phase. However, we did not find a significant association between negative affective states and the different phases of the rope-and-tie event. This suggests that all phases of roping appear to be distressing to calves.

Across all phases of the event, Evasive Behaviour and Legs Fast had an increased risk of being observed compared to Ears Axial (Table 5), implying that calf-roping events are distressing to calves. Conversely, Eye White, Legs Slow, Tail Relaxed and Vocalisation had a decreased risk of being observed. The reduced likelihood of Legs Slow and Tail Relaxed being observed indicates that calves did not experience positive mental states, as these signs associated with relaxation were less frequently observed. 

In the Release phase, most of the calves ran away from the competitor, possibly to escape further handling, explaining the presence of Legs Fast in this phase. Legs Fast was 2.6 times more likely to be observed than Ears Axial in the Release phase. Overall, calves actively attempted to escape on completion of the event. These results differed from the study by Rizzuto et al. (2020), in which calves post-roping appeared to be more relaxed, calmer and also relieved and exhausted [7]. In that study, still images rather than video footage were used, which may limit identification of behaviour, which is dynamic.

Evasive Behaviour was 3.7 times more likely to occur than Ears Axial across all phases, with the calves displaying clear signs of distress and attempting to avoid the competitor. This was evidenced by behaviours, such as calves struggling against the rope when being lassoed, caught and restrained, bucking, thrashing and in some cases struggling or attempting to stand while their legs were being tied. Conversely, six of the calves displayed little evidence of a struggle, especially during the Leg Tie phase. This may have been because calves may have been winded and/or momentarily stunned from being forced to the ground for the leg tie to occur [29]. It could also be attributed to the calves displaying signs of “learned helplessness”, where they abandon their escape attempts owing to the unavoidable nature of the capture [32]. However, it is difficult to distinguish whether the lack of behaviour exhibited by some calves during the Leg Tie phase is due to fear, learned helplessness or habituation.

The presence of both Vocalisation and Eye White were significant. We noted that Eye White was displayed predominantly in the Catch and Restraint and Leg Tie phases of the event when the calf was being directly handled by the competitor, and Vocalisation was displayed the most by calves during Catch and Restraint (Figure 1C). Goldhawk et al. found that there was increased baulking behaviour in rodeo bucking bulls associated with greater levels of human interaction [33]. Rizzuto et al. found human presence plays a significant role in welfare, as calves in rope-and-tie events appear calmer and more relaxed with fewer interactions with humans [7]. In dairy cows, aggression and dominance by stockpersons towards cattle resulted in the cattle experiencing negative behavioural states [34]. Vocalisation scoring in cattle has been used to identify welfare issues surrounding handling and procedures, such as the use of an electric prodder. Cattle vocalised less when the voltage of the device was lowered, indicating vocalisation was associated with fear and pain [35]. It has also been found that beef cattle vocalise in response to pain and in response to aversive stimuli during handling procedures [35,36]. Eye white can be used as an indicator for emotional states in cattle. For example, food-deprived cattle displayed eye white, as well as increased aggression, vocalisation and head shaking [37]. Eye white has also been observed in cows who have been separated from their calves as a sign of frustration [38]. In our study, Eye White was less commonly observed compared to other behaviours across all phases of the event. However, it was displayed predominately during Catch and Restraint and Leg Tie when the competitor was in close contact with the calf (Figure 1C,D). The presence of Vocalisation and Eye White when calves were in direct contact with the competitor suggests that physical handling in rope-and-tie events adversely affects calves.

In calves who are acclimatised to roping and transportation, it has been reported that they have no significant increase in serum cortisol concentrations after roping and transportation [39]. The absence of Evasive Behaviour in the footage of some calves could be evidence of habituation to the event; however, serum cortisol has also been reported to be increased in both naïve and experienced calves in marshalling and calf-roping events [29]. As one study suggested that acclimatisation reduced stress in cattle and another did not, further studies are needed to determine whether acclimatisation reduces stress-related behaviour in cattle and is associated with fewer changes in physiological markers for stress.

In dairy cattle, perpendicular positioning of the ears, which is similar to Ears Axial, is associated with low arousal states [40]. This behaviour was observed infrequently in the Chase and Lasso phases, where Ears Back was more commonly displayed by calves. This may be because the calves’ ears are pointed back in the direction of the competitor while being chased. Ears Axial was displayed the most in Catch and Restraint, Leg Tie and Release. As both Ears Axial and Ears Back were not significant, there is insufficient data from this study to determine the significance of ear positioning in relation to affective states in calves in the context of calf roping. 

Tail Rigid was observed the most in Catch and Restraint and the least in Release. Conversely, Tail Relaxed was observed the most in the Release phase. Tail Relaxed had significantly reduced odds of being observed (Table 5). Calves that had their tails held in a rigid manner were more common in the Chase phase of the rope-and-tie in a behavioural ethogram performed by Rizzuto et al. One explanation is that this behaviour may occur in calves as a result of being chased in a way that simulates a predator–prey interaction [7]. The decreased odds of Tail Relaxed in our study is further evidence of the rope-and-tie event being distressing to calves. 

We wanted to study Tail Swishing, as there is no published literature on this behaviour in calves in the context of rodeo as far as we are aware. Tail swishing has been observed as a frequent defensive behaviour in cattle towards irritating stimuli, such as flies [41]. In this study, it was not found to be significant across the five phases of the event; however, it occurred predominantly in Lasso, and Catch and Restraint. This may indicate that Tail Swishing is a response to the jerking forces of the rope on the calves’ neck; however, further data would be required to confirm this association. 

It was noted that one calf showed mouth gaping and tongue protrusion whilst being lassoed, caught and restrained. This behaviour has not been reported in the context of calf roping as far as we are aware. Tongue rolling and oral stereotypies have been observed in cattle who are intensively housed and are not able to display instinctual grazing behaviour [42,43]. Further work could examine if tongue positioning is an evasive or displacement behaviour in cattle used in rodeos, including calves used in calf roping. 

Independent reports about the current prevalence and severity of injury rates of calves in rope-and-tie events across all jurisdictions are not publicly available [44]. There was at least one mis-roping that occurred in one of the events we observed. The fact that mis-ropings can occur underscores the need to ensure that competitors are sufficiently experienced and competent in rope-and-tie so as not to cause additional harm to calves. While veterinarians are not required to be present at rodeos in most Australian states [45,46], the risk of injury to calves supports a need for veterinary presence at these events where they occur. This is consistent with the Australian Veterinary Association (AVA) Rodeo policy (currently under review), which states that “rodeos should be permitted only where there is appropriate legislative control to ensure the welfare of the animals involved. Such control must include a permit to operate a rodeo event underpinned by an enforceable code of practice that includes a requirement for a suitably experienced veterinarian to be involved in planning the event and to be present for the entire duration of the rodeo” [47].

Animals are sentient beings, meaning that they experience feelings and sensations, including pain and suffering [48]. This makes them subject to protection from unnecessary harm under legislation in all Australian states and territories [44]. According to the AVA, “there is considerable inherent welfare risk to animals participating in rodeos” [47]. Consequently, the AVA statement raises the concern that the welfare of the animals used is compromised by rodeos which can be conducted in a manner that is “cruel and unnecessarily dangerous”. 

Despite scientific evidence that the calves used in calf roping events experience physiological signs of stress [29], they continue to be mostly excluded from the protection from unnecessary harm, which is a fundamental requirement under animal welfare legislation across Australia. Where codes or standards are regulated under animal welfare legislation, rodeo organizers and participants are exempt from prosecution even though they treat calves in a manner which would be considered unacceptable outside a rodeo setting on welfare grounds [46].

## 5. Limitations and Future Directions

The dataset was small, footage of 15 calves in total was used in rope-and-tie events at two different rodeos. However, the sample size was sufficient to identify meaningful associations. Currently there are few published studies that are focused on the behaviour of calves in calf roping events, and given the concerns about calf roping, a prospective study may be difficult to justify ethically. This study was based on a retrospective dataset and thus the researchers had no influence on the size of the dataset.

Additionally, the quality of some footage varied, including some calf footage having a lower resolution compared to others. A limitation of the use of retrospective footage is that we could not control sound quality, and therefore there may have been instances where calf vocalisation was not audible over the noise of the crowd or loud-speaker commentary.

Physical barriers, such as fence posts, obstructed the view for some behaviours, hence they were difficult to analyse. Visual obstructions, including horses’ legs, the arena fence or dust, at times made it difficult to detect the presence or absence of some behaviours. Calf roping is a fast-paced event. As competitors have a maximum of 30 seconds, even a brief obstruction of the camera impacted scoring of behaviours.

Rodeo calf welfare may be impacted by venue-specific factors, such as calf management and handling prior to the event, noise, number of spectators, or distance calves travelled that are unique to a particular venue. Future studies evaluating larger numbers of calves at different rodeos are required to determine if there is any association between venues and negative behaviours in calves or higher prevalence of calf morbidity and mortality than others. It may be valuable to study climactic data when assessing behaviours of rodeo calves to determine how heat stress contributes to the expression of certain behaviours.

Future studies could assess the welfare of calves in calf-roping events by measuring stress biomarkers. The sympathetic adrenal medullary (SAM) response, the hypothalamic pituitary adrenal axis or haematological and biochemical markers are examples of methods that can be used to assess welfare. However, all these methods require invasive blood sampling of the animal in question and may not be practical at rodeo events [49].

Examination of calves killed or euthanized due to injury following roping events with the assistance of advanced imaging may further characterise the physical impacts on calf health and welfare.

## 6. Conclusions

Video footage was an effective tool for recording behaviours expressed by calves during a rope-and-tie event which has benefits in terms of animal welfare as it is a non-invasive and cost-effective method of assessing welfare. These rope-and-tie events elicited fear and distress in all the phases of the event for calves; and fight, flight and possibly freeze responses were observed in different calves as a response to the rope-and-tie event. Although some calves were observed to lie quietly on the ground during the Leg Tie phase, Evasive Behaviour was the most common behaviour exhibited by the calves during the event. This brings into question the justification for calf roping events to continue.

## Figures and Tables

**Table 1:** Descriptions of the five phases of calf roping used for this study, including definitions developed by the authors.

Phase	Description
Chase	The calf is chased out of the holding chute into the arena by a rider on horseback
Lasso	The rider throws a rope lasso around the calf’s neck as the calf and rider are running and then pulls the calf to a halt
Catch and Restraint	The rider dismounts, secures the calf with the rope that the calf was lassoed with and throws the calf to the ground
Leg Tie	The rider ties a knot with another rope around three of the calf’s legs to secure the calf so that it is lying in a lateral position
Release	The calf is untied and allowed to walk or trot freely towards the exit gate of the arena

Adapted from Rizzuto et al. (2020).

**Table 2:** The physical impacts and risks of injury to calves for each of the phases of calf roping.

Phase	Physical Impacts	Injury Risk
Chase	No physical contact but calf is pursued at speed by horse and rider	If the calf stumbles or falls—bruising, fractures
Lasso	Rope pulled tight around neck Calf running fast in one direction stops abruptly Calf pulled in opposite direction	Compression and damage to trachea, glands in the neck area including the thymus, thyroid, parotid salivary glands Deceleration injury where the brain contacts the inside of the skull causing concussion
Catch and Restraint	Held tightly, skin pulled, forced/dropped from about 1 m in height causing impact with ground	Bruising of skin as it is pulled and bruising/fractures to chest and ribs on contact with the ground which may temporarily “wind” the calf
Leg Tie	Whilst on the ground, three legs are forced together and tied tightly with rope	Bruising to muscles on legs and periosteum of bones in the lower legs
Release	No physical contact with competitor	Bruising, fractures if the calf stumbles or falls

Adapted from: Roy 2018 & Rizzuto 2020.

**Table 3:** Descriptions of behaviours used by observers for behaviour scoring in rodeo calves.

Behaviour	Description
Ears Axial	Ears are perpendicular to the side of the head
Ears Back/down	Ears are pointed towards the back of the animal
Eye White	Increase in the amount of sclera of the eye that is visible
Vocalisation	Calf emits noise from mouth
Leg Movement—Fast	Calf is running at full speed
Leg Movement—Slow	Calf is walking or slowly trotting
Tail Relaxed	Tail is free to move and swish
Tail Rigid	Calf is holding tail stiffly between the rear legs
Tail Swish	Calf moves the tail rapidly from side to side
Evasive Behaviour	Calf is resisting competitor (for example, pulling away at the rope, avoiding being caught or attempting to stand during Leg Tie)

(Adapted from Rizzuto et al. 2020, and Sinclair 2016).

**Table 4:** Pearson chi-squared results for each of the phases of the calf-roping event at two rodeos (*n* = 15 calves).

Phase	Pearson Chi-Square	*p* Value	Likelihood Ratio	*df*
Chase	93.083	<0.001	116.594	9
Lasso	88.666	<0.001	117.693	9
Catch and Restraint	41.408	<0.001	49.387	9
Leg Tie	24.643	<0.001	25.673	7
Release	74.574	<0.001	93.417	9

**Table 5:** Summary of the binary logistic regression analysis, which includes the behaviours of calves (*n* = 15) at two rodeos, with increased and behaviours with a decreased risk of status “1” (behaviour present) occurring. Statistically significant (*p* < 0.05) associations are highlighted in bold.

							95% CI for Exp (B)	
	B	S.E.	Wald	df	Sig.	Exp (B)	Lower	Upper
Behaviour			116.015	9	<0.001			
Ears Back	−0.517	0.341	2.304	1	0.129	0.596	0.306	1.163
**Evasive Behaviour**	**1.319**	**0.392**	**11.338**	**1**	**<0.001**	**3.740**	**1.736**	**8.060**
**Eye White**	**−1.781**	**0.392**	**20.597**	**1**	**<0.001**	**0.168**	**0.78**	**0.363**
**Legs Fast**	**0.949**	**0.393**	**5.842**	**1**	**0.016**	**2.583**	**1.197**	**5.575**
**Legs Slow**	**−1.848**	**0.425**	**18.880**	**1**	**<0.001**	**0.157**	**0.068**	**0.363**
**Tail Relaxed**	**−1.600**	**0.380**	**17.746**	**1**	**<0.001**	**0.202**	**0.096**	**0.425**
Tail Rigid	0.117	0.341	0.116	1	0.733	1.124	0.575	2.194
Tail Swish	−0.576	0.342	2.842	1	0.092	0.562	0.288	1.098
**Vocalisation**	**−1.074**	**0.354**	**9.209**	**1**	**0.002**	**0.342**	**0.171**	**0.684**
Constant	0.943	0.399	5.595	1	0.18	2.568		

## Data Availability

The data presented in this study are available on request from the corresponding author.
